# Sweet-P inhibition of glucocorticoid receptor β as a potential cancer therapy

**Published:** 2016-07-05

**Authors:** Assumpta C. Nwaneri, Lucien McBeth, Terry D. Hinds

**Affiliations:** Center for Hypertension and Personalized Medicine, Department of Physiology & Pharmacology, University of Toledo College of Medicine, Toledo OH 43614, USA

**Keywords:** Glucocorticoid receptor, GR, GR alpha, GR beta, glucocorticoids, cancer, bladder cancer, asthma, growth, migration, microRNA, miRNA, Sweet-P

## Abstract

The need for the development of new cancer therapies and push for the design of new targeting techniques is on the rise, and would be useful for cancers that are resistant to current drug treatments. The understanding of the genome has significantly advanced cancer therapy, as well as prevention and earlier detection. This research highlight discusses a potential new type of cancer-targeting molecule, Sweet-P, which is the first of its kind. Sweet-P specifically targets the microRNA-144 binding site in the 3′ untranslated region (3′ UTR) of the human glucocorticoid receptor β (GRβ), which has been demonstrated to increase expression. GRβ has been shown to be highly expressed in cells from solid tumors of uroepithelial carcinomas, gliomas, osteosarcomas, and hepatocellular carcinomas, as well as in liquid tumor cells from leukemia patients. In non-cancerous diseases, GRβ has been shown to be highly expressed in glucocorticoid-resistant asthma. These maladies brought the need for the development of the Sweet-P anti-GRβ molecule. Sweet-P was shown to repress the migration of bladder cancer cells, and may serve as a new therapeutic for GRβ-related diseases.

In our recent work, published in *Oncotarget*, we have discovered that the glucocorticoid receptor β (GRβ) causes migration (movement) of human bladder cancer cells ^[1]^. Bladder cancer is the 4^th^ most common cancer in men, and the 5^th^ most overall ^[2]^. Almost three-quarters of bladder cancer patients may have a recurrence, and one-third experience progression, causing the need for constant lifelong surveillance and treatment ^[3]^. The long-term therapy results in bladder cancer being the most costly cancer for lifetime regimen ^[4]^, which brings the need for new and better treatments. Because we showed that GRβ plays a role in bladder cancer migration, we set out to construct the first anti-GRβ molecule, which we termed Sweet-P, with the goal of providing a potential new therapy. Sweet-P was designed as a peptide nucleic acid (PNA), conjugated to the Trans-Activator of Transcription (TAT) protein from HIV (for cellular delivery) to specifically target the 3′ untranslated region (3′ UTR) of human GRβ. Sweet-P functions by specifically blocking the microRNA-144 (miR-144) binding site in the 3′UTR of human GRβ (Figure 1), which we showed increases expression. Furthermore, Sweet-P and shRNA suppression of GRβ in human bladder cancer cells attenuated migration ^[1]^.

The gene that codes for GR in humans is found on the *q* arm of chromosome 5 ^[5, 6]^, and is a single GR gene that is alternative spliced to give rise to at least five isoforms α, β, γ, A, and P ^[5, 7–9]^. GRα and GRβ have been the most investigated isoforms. GRα is identical to GRβ from exons 2–8 and is distinguished by alternative splicing of exon 9 in humans resulting in the differing of the C-terminus ^[10]^. GRα contains an additional fifty amino acids derived from the proximal portion of exon 9 that constructs helix 12 for ligand binding. GRβ does not have the capacity to bind glucocorticoids because of an additional fifteen amino acids derived from the distal portion of exon 9 that causes a degenerate helix 12 ^[5, 9, 11, 12]^. The alternative splicing mechanism in humans is different than in mouse ^[11]^, rat ^[13]^, and zebrafish ^[14]^, but in these species that GRβ has been identified, GRα and GRβ are identical through exon 8 with an addition of an alternatively spliced intron 8. In humans, the 3′ UTR of GRβ and GRα are different ^[10]^ and are targeted differently by miRNAs. For instance, miR-144 increased GRβ but had no effect on GRα expression in human bladder cancer cells ^[1]^. However, GC resistance in sepsis is influenced by miR-124, which downregulated GRα ^[15]^. The effect of miR-124 on GRβ is unknown and miRNAs that target GRβ or GRα are very limited.

GRβ has been shown to antagonize GRα, which has been demonstrated to be due to the competition with GRα for glucocorticoid response elements (GREs)/coregulators, coactivator squelching through the transactivation domain, and through inactive α/β dimers that bind in the nucleus ^[6, 11, 16, 17]^. Therefore, increasing GRβ levels can lead to a GC-resistant state that allows for an elevation of proinflammatory cytokines and transcription factors ^[10, 11, 18–20]^. The ratio of GRα:GRβ is a critical factor in GC disease states ^[10, 17, 18, 20]^. A high GRα:GRβ ratio can be indicative of a GC-sensitive state while a low ratio would be considered GC-resistant ^[18]^. Importantly, Sweet-P inhibition of GRβ increased the responsiveness to GCs ^[1]^, which indicates that it may reverse GRβ induced GC-resistant diseases. Also, GRβ has recently been shown to have positive and negative GRα independent transcriptional activity ^[6, 12]^. We recently demonstrated that mouse GRβ specifically binds to the promoter of phosphatase and tensin homolog (PTEN), which increased Akt1 guided proliferation ^[21]^. We also showed that Sweet-P inhibition of human GRβ increased PTEN expression in bladder cancer cells ^[1]^ (Figure 1). There may be other GRβ-specific gene targets that are increased in cancer, and microarray or RNA-seq studies would help strengthen our understanding of the involvement of GRβ in cancer. However, this work is yet to be done.

Sweet-P may have several clinical applications as GRβ has been shown to be involved in other cancer types. For example, treatment with GCs as a first line therapy in acute lymphoblastic leukemia (ALL) is effective due to its ability to arrest cell growth and trigger apoptosis. Unfortunately, resistance to therapeutic GCs is common, which has been attributed to increased levels of GRβ or decreased GRα ^[22]^. The GRβ interaction with β-catenin and transcription factor-4 (TCF-4) was shown to positively regulate astrocyte activity, leading to increased proliferation ^[23, 24]^. This observation further supports our previous finding of GRβ stimulation of growth ^[21]^, albeit via Akt1 activation and PTEN inhibition. Also, GRβ was shown to increase migration of glioblastoma cells ^[25]^. However, the interaction of miR-144 with the GRβ 3′UTR in glioblastoma or ALL is unknown. In LNCaP-ARA70β prostate cancer cells, which express increased levels of GRβ, Ligr *et al.* reported increased cellular growth and proliferation ^[26]^. Furthermore, treatment with methotrexate in peripheral mononuclear and lymphocyte cells resulted in decreased GRβ expression, thus increasing GC sensitivity ^[27]^. Additionally, Piotrowska *et al.* demonstrated in Hut-78 and Raji B-lymphoma cells, MCF-7 breast cancer cells, and HT-29 colon carcinoma cells that known growth inhibitors trichostatin, sodium butyrate, and 5-Aza-20-deoxycytidine treatment suppressed GRβ and enhanced GRα with an increase in GC sensitivity ^[28, 29]^. However, miR-144 levels, proliferation, or migration were not assessed in these studies. Nevertheless, these observations indicate the necessity of developing an anti-GRβ therapy to specifically target GRβ-related cancers.

In non-cancerous diseases, the resistance to GCs due to high levels of GRβ have been reported, and Sweet-P can potentially be used as a novel therapy. Most clinically relevant is the anti-inflammatory and immunosuppressant effects of GCs, which have been shown to decrease levels of cytokines, chemokines, and vasoactive agents. GCs reduce the movement of leukocytes to inflamed areas, and the function of immunocompetent cells ^[8]^. In mice, increased GC levels induce apoptosis in thymocytes ^[30]^. Because of the anti-inflammatory effects of GCs, they are commonly prescribed to asthma patients. Many studies have demonstrated an elevated expression of GRβ and GC-insensitivity in the airways of asthma patients ^[31–34]^. Christodoulopoulos *et al.* showed that approximately 8% of cells in large and 2% of cells in small airways of patients were GRβ positive. However, mild asthma patients had an increase of 14% (7 fold) in GRβ positive cells in the small airways, but no change in expression in large airways. Alarmingly, in fatal asthmatic patients, the airways showed a dramatic increase in GRβ positive cells to 21% (2.5 fold) in large and 35% (17 fold) in small airways ^[31]^. Hamid *et al.* reported an increased number of GRβ immunoreactive inflammatory cells in the airway T-cells of GC-resistance patients when compared to GC-sensitive or healthy patients ^[33]^. In tuberculin-driven cutaneous inflammatory lesions of patients with GC-resistance asthma, increased number of cells expressing GRβ was also reported ^[34]^. Furthermore, Goleva *et al.* demonstrated in bronchoalveolar lavage macrophages that GC-insensitive asthmatics have elevated GRβ mRNA and protein levels in comparison to GC-sensitive patients ^[32]^. Of most interest, the authors reported enhancement of dexamethasone-induced GRα transactivation in GC-insensitive asthmatics after RNAi silencing of GRβ. As such, Sweet-P suppression of GRβ may serve useful for GC-insensitive asthmatic patients.

Our work highlights miR-144’s role in inducing migration of bladder cancer cells via GRβ; however, miR-144 has been demonstrated to play roles, both positive and negative, in many other forms of cancers and diseases. For example, miR-144 has been shown to contribute to the pathogenesis of Alzheimer’s disease through the downregulation of ADAM10 ^[35]^ but is essential for proper erythropoiesis by downregulating RAB14 ^[36]^. Also, miR-144 has been shown to promote nasopharyngeal carcinoma through the downregulation of PTEN, a regulator of the PI3K/AKT pathway ^[37]^, and induce breast cancer and hepatocarcinoma cell proliferation through the downregulation of Runx1, a tumor-suppressor gene ^[38]^. Interestingly, estrogen treatment (E2) in SkBr3 breast cancer and HepG2 hepatocarcinoma cells increased the expression of miR-144 through the PI3K/ERK/Elk1 transduction pathway ^[39]^, which may serve as a positive activator of GRβ. Solakidi *et al.* showed in HepG2 and SaOS-2 cells that GRβ and ERα were localized mainly in the nucleus, particularly concentrated in nuclear structures which suggest a direct involvement of GRβ and ERα in nucleolar-related processes ^[40]^. However, the interaction of ERα and miR-144 signaling to increase GRβ activity has not been studied. In contrast to oncogenic properties of miR-144, the loss of miR-144 expression has been shown to be related to the progression of colorectal cancer through the derepression of mTOR, a cell growth and metabolism regulator ^[41]^. However, because the decrease in miR-144 expression leads to colorectal cancer progression, GRβ may not have an involvement. Also, miR-144 has been shown to inhibit the migration, invasion, and proliferation of carcinomas such as rectal cancer ^[42]^ and osteosarcoma ^[43]^, which was attributed to the downregulation of ROCK1 ^[42]^. High levels of GRβ was shown in SaOS-2 osteosarcoma cells, which suggest that miR-144 and GRβ signaling may be differentially regulated in bone cancer. Similarly, miR-144 inhibited migration and proliferation of hepatocarcinoma cells by the downregulation of AKT3 ^[44]^ and non-squamous cell lung carcinoma through the downregulation of ZFX ^[45]^. Due to the diverse targeting of many different genes, inhibiting the global function of miR-144 during cancer therapy could be detrimental through off-target effects, and result in the de-repression of oncogenes. The specificity of Sweet-P blocking only the interaction of miR-144 with the 3′UTR of GRβ (Figure 1) to suppress cancer cell migration may be particularly useful due to the presentation of fewer side effects.

In conclusion, our discovery of Sweet-P targeting GRβ in bladder cancer sheds light on a novel drug therapy that specifically targets a gene known to cause growth, proliferation, migration, and GC hormonal therapy resistance. At this point, we have shown that the Sweet-P molecule suppresses GRβ in bladder cancer. In addition, we have shown that Sweet-P only targets GRβ and not other miR-144 regulated genes. More importantly, Sweet-P inhibits the ability of cancer cells to migrate. We will also be testing the effect of the Sweet-P molecule on other types of cancer. Essentially, Sweet-P may be used as a treatment option for several different carcinomas where GRβ is highly expressed including bladder, prostate, lung, or glioblastoma, as well as for liquid tumors such as in leukemia. Sweet-P can be beneficial for non-cancerous diseases also, such as asthma and GC-insensitive disease states caused by increased GRβ. Thus, Sweet-P serves as the first anti-GRβ molecule that may provide a new therapy.

## Figures and Tables

**Figure 1:**
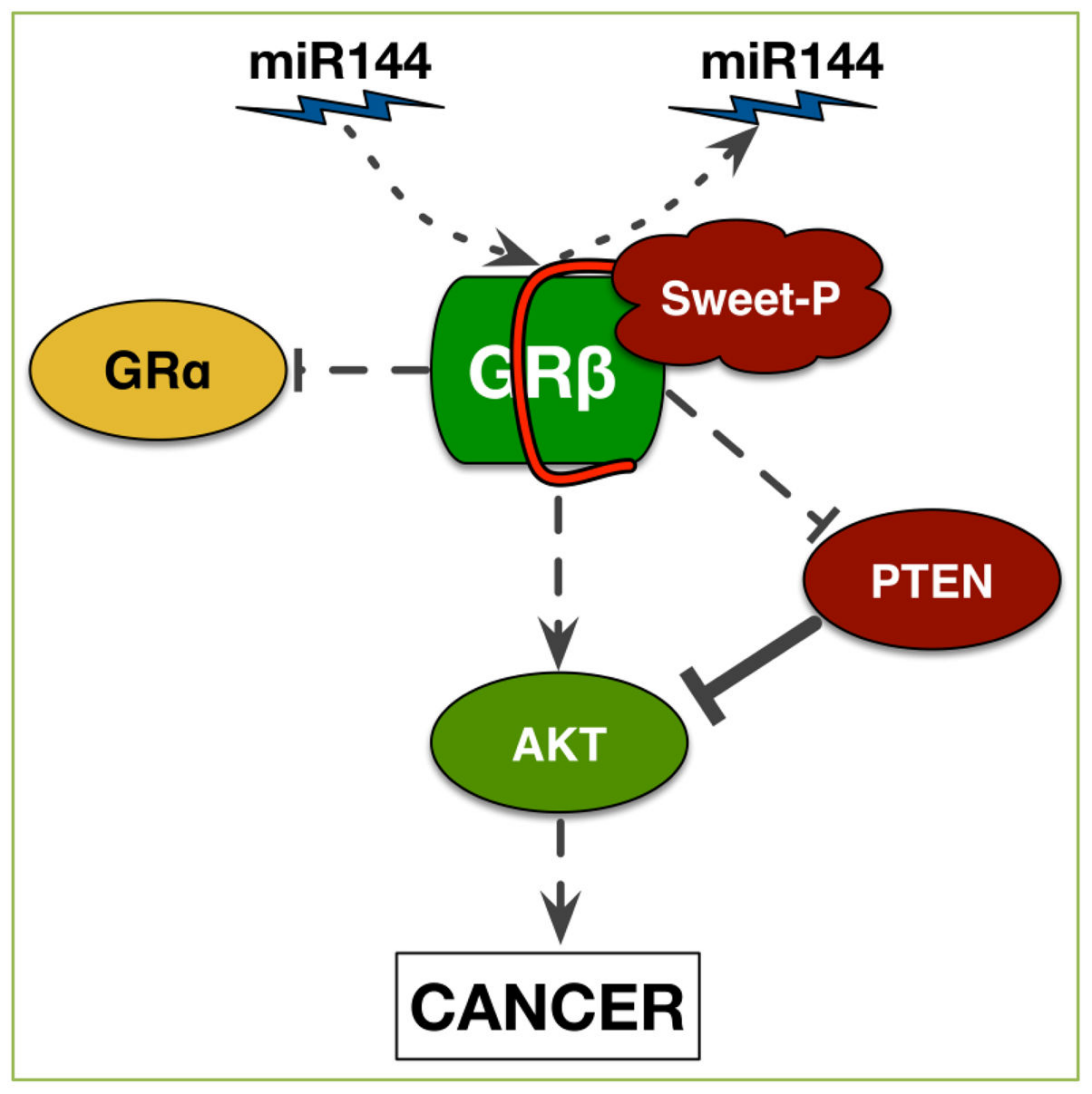
Sweet-P inhibition of GRβ reduces signaling that leads to cancer Sweet-P inhibits miR144 binding to the 3′UTR of human GRβ, resulting in reduced expression. Sweet-P inhibition of GRβ increases GRα and PTEN activity and decreases AKT, which leads to reduced cancer growth and migration.
